# Recent progress in genomic prediction for Hanwoo cattle and its implications for beef quality: review

**DOI:** 10.5713/ab.250562

**Published:** 2025-12-03

**Authors:** Monira Akter Mou, Md Azizul Haque, Jong-Joo Kim

**Affiliations:** 1 Department of Biotechnology, Yeungnam University, Gyeongsan, Korea

**Keywords:** Beef Cattle, Genomic Best Linear Unbiased Prediction, Genomic Selection, Hanwoo, Prediction Accuracy

## Abstract

Genomic selection (GS) has become an indispensable tool in the beef cattle industry, offering the potential to significantly enhance genetic gain and prediction accuracy by integrating genomic, pedigree, and phenotypic information to estimate genomic breeding values. Hanwoo cattle (HC) are valued in the Korean Peninsula, for their exceptional marbling and distinct flavor. Thus, genetic improvement breeding programs for Hanwoo have been undertaken to improve beef production and profitability, emphasizing particular focus on carcass and meat quality traits. The success of a breeding program that incorporates genomic information in HC is largely dependent on prediction accuracy, thereby making genomic prediction (GP) essential for accelerating genetic gain. Hence, breeders must recognize the superiority of GS and choose the most suitable prediction model based on the genetic architecture and biological nature of the trait of interest. Several GP methods have already proven superior performance regarding carcass and meat quality traits compared to the traditional pedigree-based best linear unbiased prediction method. Consequently, no alternative approaches exist for breeders to accelerate the innovative development of Hanwoo beef cattle to GS. The main objective of this review is to provide an overview of the application of GP methods in improving the ultimate meat quality of HC. Furthermore, this review presents the transversal analysis of interest in GS for Hanwoo breeders regarding the selection of best prediction methods for specific traits. This review literature is expected to serve as a significant resource for future exploitation of Hanwoo's potential in the Korean beef industry.

## INTRODUCTION

Hanwoo is the most popular beef cattle breed in Korea and is prized for its excellent marbling, tenderness, juiciness, and unique flavour. It is a native taurine breed of Korea that was historically used as a draft animal, dating back 5,000 years [1]. Traditionally, it has been utilized in farming, transportation, and religious ceremonies, but it has undergone a significant transformation over the past 30 years into a specialized meat-type breed [2]. This shift was driven by the rapid economic growth of Korea in the 1960s, which increased beef consumption and supported commercial Hanwoo production [1].

**Figure 1:**
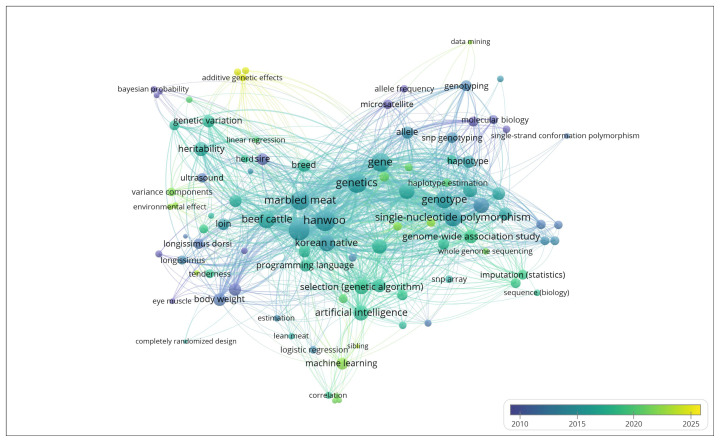
Review literature of Hanwoo cattle focusing on meat quality.

Korean consumers have valued Hanwoo beef for its flavour, tenderness, marbling, and health benefits, including low cholesterol and high omega-3 content, which are associated with a lower risk of cardiovascular disease [3], offering a healthier alternative than other beef breeds. As a result, this economically important breed has become the focus of extensive research aimed at improving both meat quantity (protein content) and quality (taste and health consciousness of meat). Thus, carcass traits, including carcass weight (CWT), backfat thickness (BFT), the eye muscle area (EMA), the marbling score (MS), and the rib eye area (REA), have been prioritized as selection criteria in commercial Hanwoo breeding programs. Since the 1960s, HC has undergone remarkable genetic improvements, resulting in exceptional enhancements in meat quality, which makes it one of the world’s leading sources of premium, highly marbled beef [4]. Early genetic improvement efforts focused mainly on carcass quality and growth traits because of the availability of trait information and simple analytical tools [5]. Figure 1 illustrates the major research themes and keywords from the literature on HC, highlighting the long-term scientific focus on meat quality and carcass improvement. Factually, carcass traits (CWT, BFT, EMA, and MS) are quantitative traits that are moderately to highly heritable [6,7] and polygenic in nature and are controlled by multiple genes with small-effect SNPs [8,9]. Despite their economic importance, owing to their direct influence on meat yield, carcass quality grade, and overall profitability, these traits are difficult to measure in live animals [10], thereby emphasizing the necessity of GS to achieve more accurate and efficient genetic improvement.

Historically, breeding programs relied on pedigree and phenotypic information to identify superior animals. The estimation of breeding values (EBVs) using the best linear unbiased prediction (BLUP) method developed by Henderson [11] represented a fundamental step in genetic evaluation. When direct phenotyping of selection candidates is impossible (such as with lethal traits or sex-linked traits), breeding values are traditionally predicted via data from relatives (full or half-sibs) or pedigree records. Without genomic information, traditional methods provide the same breeding value to sibs, ignoring within-family genetic differences. Interestingly, the advent of GS addressed this limitation by utilizing genome-wide SNP information to distinguish individual genetic differences. Consequently, GS has since become a cornerstone of modern animal breeding, offering enhanced accuracy, efficiency, and genetic gain across species and traits by leveraging dense genomic information [12]. Moreover, GS enables a significant decrease in the generation interval by accelerating the accuracy of breeding in young animals [13]. With the introduction of high-density SNP panels and thousands of identified markers, the prediction of genetic merit through genomic estimated breeding values (GEBVs) became more reliable [14]. Moreover, GS could increase accuracy by reducing the need for phenotypes for different species when traits are difficult or costly to measure with low heritability [15] or strong genotype-environment interactions [16]. Initially, the adoption of GS was limited by the high cost of genotyping; however, as costs continue to decrease, its use has expanded [17]. On the other hand, fully replacing phenotyping with genotyping shortens the breeding cycle by enabling early selection without waiting for phenotypic data [18]; partial phenotyping replacement filters out poor candidates, leading to increased selection intensity [19]; and finally, the remaining candidates are evaluated with both genomic and pedigree information at no extra cost [17]. Furthermore, genomic information helps to select parents with high breeding values with complementary traits, resulting in improved progeny quality and providing more precise genomic relationships than pedigree relationships, aiding in maintaining genetic diversity. Therefore, interest in the use of GP methods has also increased among the beef industry. Importantly, various genomic evaluation approaches have shown similar levels of prediction accuracy [20] but with higher accuracies than the traditional pedigree-based best linear unbiased prediction (PBLUP) [21,22].

**Figure 2:**
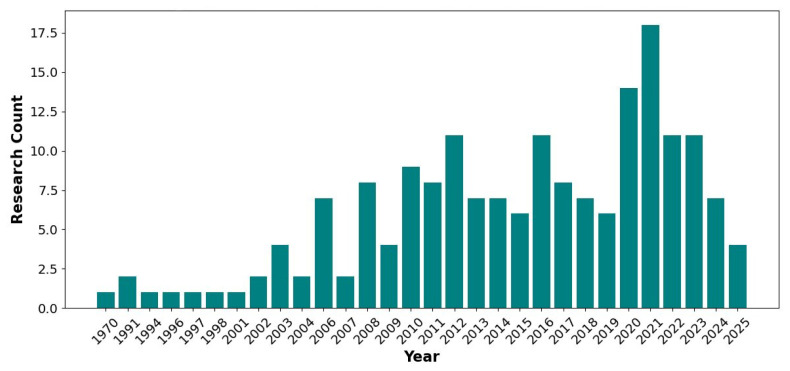
Yearly research contribution for carcass traits in Hanwoo.

**Figure 3:**
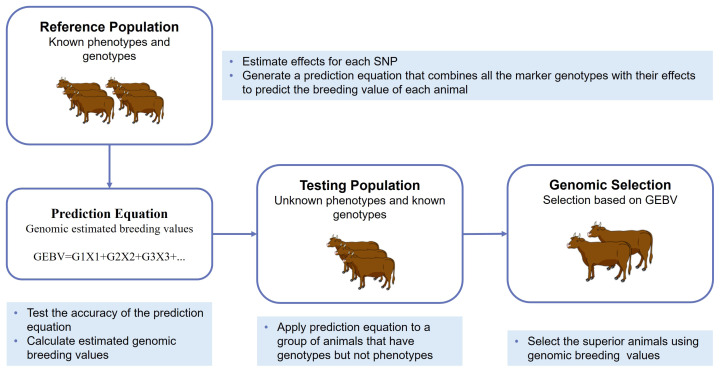
Genomic selection procedure. GEBV, genomic estimated breeding value.

In Hanwoo, recent efforts have focused on implementing GP methods to improve breeding programs and enhance economically important traits, particularly carcass traits, which are difficult to measure in live animals. Research contributions from 1970 to 2025 have demonstrated the rapid development of statistical methods in Hanwoo breeding (Figure 2). However, studies found positive genetic correlations among Hanwoo carcass traits that enable simultaneous improvement, making them ideal for GS-based selection [23,24]. The genomic models that utilize SNP information can effectively capture Mendelian sampling effects [25], which helps make breeding predictions more accurate. Furthermore, the integration of high-density SNP arrays and multiomics data has contributed to more accurate GEBVs for key carcass traits [8,23,26,27]. Empirical evidence supports the effectiveness of GS in Hanwoo; for example, Seo et al [28] reported annual genetic gains of 0.78 kg in yearling weight, 0.35 kg in CWT, and 0.27 cm^2^ in the EMA. Moreover, between 2000 and 2016, the average CWT of Hanwoo steers rose from 343 kg to 437 kg, alongside an improvement in MS from 3.6 to 5.6 [29], reflecting substantial progress in meat quality. This continued genetic progress encourages Hanwoo breeders to place greater emphasis on the adoption and expansion of GS strategies. Despite significant progress, there are still several challenges observed in the application of GS to the Hanwoo breeding program. The limited size of the Hanwoo reference population continues to constrain the accuracy of genomic evaluation. In addition, integrating multi-omics data (such as transcriptomics, metabolomics, and epigenomics) into breeding programs is still underdeveloped. Researchers also face difficulties in applying advanced statistical and computational methods, particularly machine learning (ML), due to data complexity, heterogeneous trait architecture, and the need for large, well-structured datasets. Addressing these gaps is crucial for maximizing the benefits of genomic advances in Hanwoo breeding. The overall framework of the GS procedure, encompassing reference population establishment, genotyping, prediction og GEBVs, and selection of superior breeding animals, is illustrated in Figure 3. In recent years, genomic best linear unbiased prediction (GBLUP) and Bayesian approaches have been widely adopted to increase the prediction accuracy of Hanwoo over PBLUP [30–33]. Moreover, ML has also emerged as a promising approach in the genomic era, because of their tremendous flexibility and ability to model complex and nonlinear patterns in high-dimensional and noisy genomic datasets [34], where linear models most often face statistical challenges and show poor performance [35,36]. For example, breeders want to increase MSs; in such cases, the ML model, particularly deep learning, could be a highly promising prediction model because it could handle the challenges in Marbling [37].

Overall, this review synthesizes the current advances in GP models applied to Hanwoo cattle, highlighting the key milestones in breeding strategies and improvements in the predictive accuracy of carcass traits. Our study underscores the need for continued development and refinement of GS programs to achieve sustainable genetic progress by enhancing economically important traits while preserving superior meat quality. GS remains the most powerful and efficient framework to achieve these objectives, enabling accelerated genetic gain through accurate estimation of genomic breeding values. Moving forward, expanding reference populations, integrating multi-omics data, and optimizing ML algorithms will be indispensable for further improving the accuracy, robustness, and long-term sustainability of genomic evaluation systems in Hanwoo beef cattle.

## GENOMIC PREDICTION METHODS

**Figure 4:**
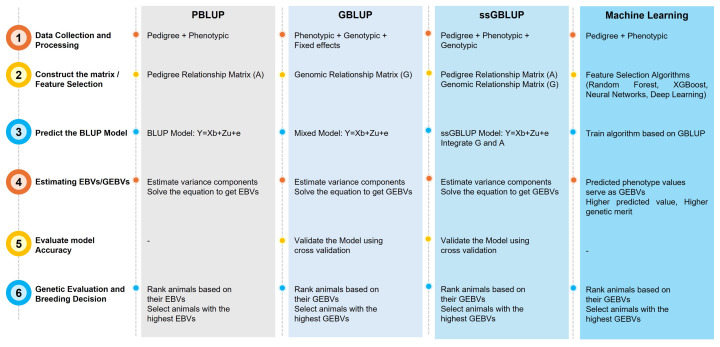
Breeding value estimation using different methods in Hanwoo cattle. PBLUP, pedigree-based best linear unbiased prediction; GBLUP, genomic best linear unbiased prediction; ssGBLUP, single-step GBLUP; GEBV, genomic estimated breeding value.

Several animal-based statistical models and ML algorithms have been developed with the introduction of the GS concept over the traditional method to strengthen genetic gains by selecting superior animals early in life or even before birth. However, an essential fact in GP is the selection of a suitable statistical method, which is reliant upon trait complexity, population structure, and resource processing. The fundamental disparities among the statistical models that have been put forth thus far are found in the assumptions that they make about the distributions of SNP effects and the genetic architecture underlying the traits [38]. In addition, a major challenge arises when the number of markers greatly exceeds the number of observations, leading to limited degrees of freedom and more variables than with individuals [39]. Notably, such challenges could be addressed by using SNP-BLUP, GBLUP, Bayesian regression models, and ML algorithms. However, the prediction through EBVs and GEBVs in Hanwoo cattle is factually coordinated by the National Institute of Animal Science (NIAS) under the Rural Development Administration (RDA), Korea. NIAS operates the Hanwoo progeny testing and improvement program, which collects performance, pedigree, and genomic data from nationwide herds. The system provides a structured framework for the selection of superior artificial insemination (AI) bulls and replacement heifers based on genetic merit. Moreover, EBVs or GEBVs estimation for carcass and growth traits forms the foundation of the official Hanwoo selection index used in national breeding programs. The estimation of the breeding value of HC via different methods is illustrated in Figure 4. Some of the most common approaches applied in the Hanwoo breeding program, along with traditional methods, are briefly discussed in the following sections:

### Pedigree-based traditional model

PBLUP, which utilizes the numerator relationship matrix (A), is traditionally applied to estimate EBVs based on an animal’s own and progeny phenotypes [40]. The general PBLUP model [23]:


(1) y=Xb+Za+e

where *y* is the vector of observations; b is the vector of the fixed effects; a is the vector of the additive genetic effects; and e is the vector of the residuals. In contrast, X and Z are incidence matrices related to fixed and additive genetic effects, respectively.

In HC, genetic evaluation is carried out through the Hanwoo Performance and Progeny Test program using pedigree-based BLUP or genomic BLUP. In this system, Lee et al [1] employed a pedigree-based BLUP approach to estimate EBVs using performance and carcass data collected from young bulls and progeny steers. The multi-trait animal model revealed a positive genetic correlation between EMA and CWT, and a negative correlation between BF and MS. Notably, Pedigree-based BLUP has contributed to steady genetic improvement in Hanwoo carcass traits; however, its reliance on pedigree and phenotypic data limits prediction accuracy for young animals, leading to slower genetic gain compared to GS [1,41].

### Genomic best linear unbiased prediction

GBLUP estimates GEBVs via a genomic relationship matrix (G) derived from DNA marker data instead of an A matrix [42]. The GBLUP model applied to Hanwoo [43] is as follows:


(2) $$y=X_{b}+Z_{u}+e$$

where y is the vector of phenotypes; X is the incidence matrix of b; b is the vector of fixed effects; Z is the design matrix assigned to u; u is the vector of individual additive genetic effects, and e is the vector of random residual effects on the phenotype.

Recent evaluations have implemented GBLUP and its derivatives, like single-step GBLUP (ssGBLUP), weighted GBLUP (WGBLUP), and weighted ssGBLUP (WssGBLUP) in Hanwoo, which integrates pedigree, genotypic, and phenotypic records through the H matrix, combining the traditional A matrix and the genomic G matrix model. The incorporation of genomic information through GBLUP has yielded noticeable improvements in the estimation of GEBV accuracy, outperforming conventional PBLUP by 12% to over 53% [1,25,31,44,45]. Using the ssGBLUP approach, Lee et al [46] achieved high prediction accuracies for carcass traits (0.73–0.77), while Jang et al [43] reported realized accuracies up to 0.78 for MS and 0.73 for CWT using a large reference population combining cows and steers. Both of the studies integrated pedigree (A) and genomic (G) relationships into the combined H matrix to simultaneously evaluate genotyped and non-genotyped animals, where BLUPF90 family and ASReml were used, respectively, for variance component estimation and solving mixed model equations. Moreover, multi-trait ssGBLUP using the H matrix consistently achieved the highest prediction accuracies for carcass traits, particularly when correlated ultrasound or yearling weight records were included [31,47]. This model outperformed pedigree-based BLUP, yielding the highest accuracies, especially for CWT (0.69–0.70) and MS (0.71–0.76). These evaluation studies indicated that CWT and MS are the most suitable traits for GP using the ssGBLUP model in Hanwoo cattle. Therefore, the main strength of this model is its improved accuracy and efficiency in estimating breeding values for carcass traits, more pertinently, when multi-trait models incorporate correlated indicators such as ultrasound and yearling weight.

To increase predictive power, WGBLUP was introduced, which enhances predictive performance by incorporating locus-specific variances through differential SNP weights on the basis of their effect sizes in the construction of the G matrix [48]. However, across Hanwoo genomic evaluation studies, WGBLUP has reported contradictory results depending on dataset composition and trait genetic architecture. Lopez et al [49] applied WGBLUP, assigning greater weight to SNPs with larger effects, where both linear and nonlinear iterative weighting approaches were tested for ten iterations using BLUPF90 software. However, they observed only minor accuracy gains (2%–5%) of WGBLUP over GBLUP, particularly for CWT, whereas there was no clear advantage for MS. In contrast, Haque et al [50] reported a notable improvement (up to 9%) by iterative WGBLUP over GBLUP, with the highest accuracy for MS (0.86), where a larger dataset and multi-iterative WGBLUP were applied to capture heterogeneous SNP variances to evaluate carcass traits of Hanwoo. This discrepancy likely reflects differences in reference population size, SNP density, and weighting algorithms, indicating that WGBLUP performance in Hanwoo is dataset and trait-dependent. Importantly, WGBLUP significantly captures heterogeneous SNP effects, improving accuracy for complex carcass traits and outstriping both GBLUP and Bayesian alphabets while remaining computationally efficient for national-scale application [43,50,51]. Despite improvements, both weighted and non-WGBLUP have been found to yield less accurate and biased predictions when pedigree or phenotypic data from non-genotyped animals are missing [52]. To overcome this, WssGBLUP was proposed, which combines the strengths of ssGBLUP and WGBLUP for more accurate genomic predictions [48]. Mehrban et al [53] applied WssGBLUP to over 21,000 Hanwoo animals, achieved higher prediction accuracies, particularly for CWT (0.69–0.70) and MS (0.74–0.76), which outperformed both PBLUP and ssGBLUP. Notably, the accuracy and stability of GBLUP and its derivatives are heavily dependent on reference population size, marker density, and data quality; when these are insufficient, the model may produce biased or less consistent predictions across herds and generations.

### SNP-best linear unbiased prediction

SNP-BLUP estimates GEBVs directly from individual SNP effects via a marker-based model rather than a genomic relationship matrix, and is an alternative to GBLUP, which uses a genomic relationship matrix for predicting breeding values [45]. The SNP-BLUP method, also termed random regression or ridge regression BLUP [42], considers these SNP effects and breeding values, respectively, as random effects [54]. The general form of the SNP-BLUP model [55] is as follows:


(3) y=Xb+Zu+e

where *y* is the vector of phenotypic observations; *X* is the design matrix for fixed effects; *b* is the vector of fixed effects; *Z* is the design matrix for random effects; *u* is the vector of random SNP effects; and *e* is the vector of random residual effects.

SNP-BLUP has been applied to Hanwoo cattle to predict breeding values for major carcass traits (CWT, EMA, BF, and MS) using large-scale national datasets managed by KAIA (Korea Animal Improvement Association) and KIAPE (Korean Institute for Animal Products Quality Evaluation). Accordingly, Kim [56] implemented an SNP-BLUP REL approach in MiX99 and custom FORTRAN code to compute EBV directly from SNP effects. Moreover, Koo et al [57] expanded this to a single-step SNP-BLUP (ssSNP-BLUP) that integrates over 8 million carcass records and almost 30 thousand genomic information of Hanwoo in one framework, allowing simultaneous prediction for genotyped and non-genotyped animals. However, both studies found higher prediction accuracy and lower bias compared with pedigree-based BLUP, particularly in large-scale, multi-year datasets. More specifically, CWT showed the highest prediction accuracy, indicating it is the most suitable trait for SNP-BLUP-based genomic evaluation in Hanwoo [56,57]. Furthermore, SNP-BLUP may have lower accuracy than Bayesian methods, particularly with small sample sizes and high marker density [58]. More importantly, these studies highlighted the computational efficiency and the ability of the SNP-BLUP model to directly estimate marker effects as key strengths, while noting that its accuracy depends on SNP density and reference population size, and that it may overlook the effects of small-effect loci.

### Bayesian regression model methods

Bayesian regression methods have gained popularity because of their versatility in stimulating a wide range of genetic architectures, which makes them ideal for traits influenced by quantitative trait loci (QTLs) with moderate to large effects. Unlike GBLUP, which assumes equal variance across all SNPs, Bayesian models assign variable effects and shrinkage levels on the basis of prior distributions. However, all Bayesian multiple regression models are basically described [59] by the following equation:


(4) $$y=\mu +\sum_{k=1}^{m}x_{k}\beta_{k}+e$$

where *y* is the vector of pre-corrected phenotypes, *μ* is the overall mean, *x_k_* is the vector of genotypes for the k^th^ SNP, *m* is the number of SNPs, *k* is the effect of the k^th^ SNP, and *e* is a vector of random residuals.

Recent studies have applied Bayesian approaches in GenSel or GS3 software to estimate GEBVs for Hanwoo carcass traits, where GEBVs were obtained by summing the posterior mean SNP effects weighted by genotypes. Bayesian approaches (particularly BayesC, SSBR) consistently outperformed GBLUP for CWT, whereas for BFT, EMA, and MS, Bayesian methods perform similarly to GBLUP, reflecting the polygenic nature of these traits [50,52,60]. Additionally, WGBLUP accuracies were 8.97% higher than the GBLUP and 1.80% higher than the Bayesian alphabets on average [50]. More pertinently, these studies indicated that Bayesian methods are most suitable for traits influenced by a few major QTLs like CWT, alongside numerous small-effect loci. However, a major limitation is that model performance depends on the genetic architecture of each carcass trait, meaning that a method optimal for one trait may perform poorly for another [60].

### Machine learning algorithms

**Figure 5:**
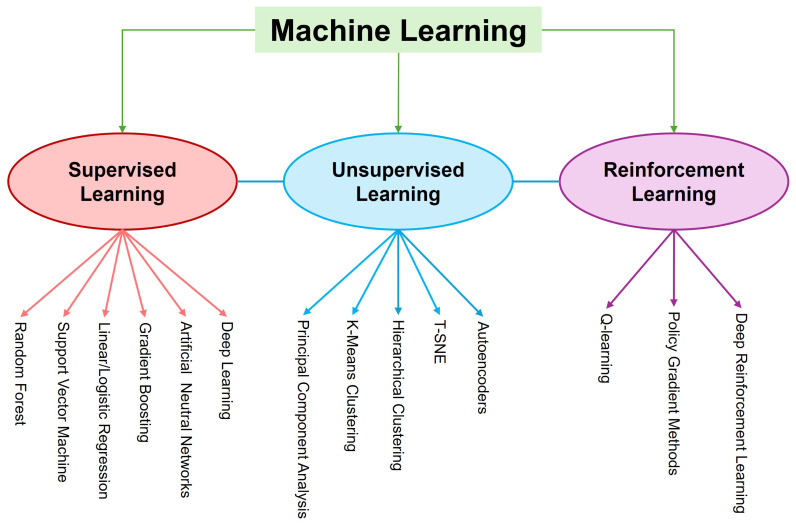
Machine learning algorithms classification.

In animal breeding, ML is an advanced approach that leverages artificial intelligence algorithms to analyze large datasets and improve breeding programs. Arthur Samuel [61] first coined the term ‘machine learning’, which has a long history that dates to 1959. Traditional linear models often struggle with issues such as collinearity, high dimensionality, and nonlinear relationships, whereas ML algorithms generally perform better in handling noisy and high-dimensional genomic datasets to predict the GEBVs in animals [36]. In the learning process, ML algorithms can be categorized into three types of learning, namely, supervised learning, unsupervised learning, and reinforcement learning (Figure 5). Notably, the supervised learning technique is the most widely used ML technique in GP [34]. Recently, a few studies have extensively focused on applying ML to predict Hanwoo carcass traits; however, none have outweighed GBLUP in overall accuracy or robustness [7,62]. They claimed GBLUP remains the preferred approach for genomic breeding value prediction, as it provides unbiased additive genetic estimates, is easily interpretable within existing evaluation systems, and remains stable even with moderately sized reference populations. For instance, Srivastava et al [7] aimed to explore whether ML models, namely, RF, XGBoost, and SVM, could outperform GBLUP in predicting GEBVs for key carcass traits in HC. Their goal was to test whether these non-linear models can capture complex genetic architectures that the traditional linear GBLUP model might miss, thereby improving the accuracy and efficiency of GS in Hanwoo cattle. Although the ML methods demonstrated potential for capturing non-linear genotype–phenotype relationships, they did not outperform GBLUP in overall prediction accuracy. Furthermore, another study [62] developed a GP framework for Hanwoo carcass traits using ML algorithms (SMO, RF, MT, MLP) instead of conventional GBLUP. They aimed to evaluate their ability to handle unbalanced trait distributions through synthetic data augmentation and suggested that ML can be a promising complementary approach for GP in Hanwoo, especially for complex, non-linear traits such as MS. Despite the significant flexibility in modeling complex relationships, the routine adoption of ML Hanwoo breeding remains constrained by population size, data quality, and interpretability challenges. However, expanding the genomic reference population and hybrid frameworks combining statistical and ML models like deepGBLUP [63] could enhance genetic gain in the Hanwoo breeding program.

## HANWOO BREEDING INITIATIVE FOR IMPROVEMENT

**Figure 6:**
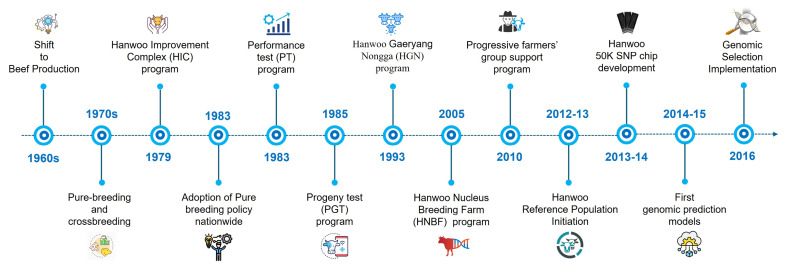
Key breeding strategy of Korean Hanwoo cattle.

Figure 6 depicts the key milestones of the Korean HC breeding strategy [1,4]. In the 1960s, with the rapid growth of the Korean economy, meat demand surged, prompting the government to establish the Livestock Industry Act to guide Hanwoo improvement as a beef breed. In 1969, the first National Hanwoo Championship was held, and three proven bulls were selected for a nationwide AI program. During the 1970s, farmers’ strong interest led to efforts focused on increasing body weight through both crossbreeding and pure breeding. However, crossbreeding has failed to improve beef quality or reduce production costs compared with imports. As a result, it was discontinued, and the focus shifted entirely to pure breeding, emphasizing both qualitative and quantitative improvements in meat traits. In 1979, the first national breeding program, the Hanwoo Improvement Complex (HIC), or *Hanwoo Gaeryang Danji*, was launched by the Ministry of Agriculture and Forestry (MAF) to promote purebred cow-calf production. Nevertheless, HIC remained unimpressive and was stopped in 1998 because of limited farmer involvement, poor record-keeping, and a shortage of skilled staff. In 1983, cow-calf farms were established, and the pure-breeding policy was widely adopted. As a follow-up initiative, the Hanwoo proven bull selection program was introduced in two stages: i) a performance test (PT) in 1983 and ii) a progeny test (PGT) in 1985 to improve the body growth rate and meat quality. This led to the first selection of 10 proven bulls in 1987. The Hanwoo improvement farm (HIF), or *Hanwoo Gaeryang Nongga*, was established in 1993, similar to the HIC, but focused on individual farm evaluations rather than a group of farms together. After a successful 5-year pilot experiment, HIC was replaced, and HIC was continued until 2009. However, short breeding windows, unstructured mating plans and dam selection, minimal cow participation, and inadequate emphasis on cow performance beyond pedigrees and appearance made it difficult for both the HIC and HIF programs to successfully support the Hanwoo proven bull program. Consequently, the MAF introduced a major amendment in 2004 to improve cow-calf farm management, with a particular focus on performance recording, seasonal breeding, and disease control. Following this great initiative, the new Hanwoo Nucleus Breeding Farm program, or *Hanwoo Yookjong Nongga*, started in 2005 and achieved significant success with the establishment of 104 nucleus farms. In 2010, after HIF ended, the MAF launched the Progressive Farmers’ Group Support program. It supported farmers who invested in livestock improvement and required payments for specific services (ultrasound scans, animal registration, and breeding consultation). A major improvement was the inclusion of comprehensive consulting services, including genetic evaluation and mating plans, offered by 11 expert institutions authorized by NIAS, ensuring consistency and quality, an area where the HIF was lacking. In Hanwoo, pedigree-based selection achieved gradual improvement in breeding objective traits such as CWT and EMA with a slight negative genetic response (−0.036) of MS per year [1,41]. Besides, the prediction accuracy of EBVs is low to moderate (Table 1), which largely depends on pedigree depth, record quality, and herd size [41]. However, traditional breeding methods are often inaccurate and insufficient involving, long generation intervals, slow genetic gains, and low prediction accuracy, particularly for low-heritability traits such as MS. These limitations were addressed with the advent of genome-wide SNP panels, leading to the development of a reference population with high-density SNP genotypes between 2012 and 2013. Despite this, NIAS and RDA developed the Hanwoo 50K SNP chip for the application of genomic selection to HC. The first GP models were subsequently trained on reference population data from 2015 and subsequently applied to the carcass traits of Hanwoo. Since 2016, broader GS adoption in the Hanwoo breeding program has started. This transition of Hanwoo to GS aimed to increase prediction accuracy, accelerate genetic gain, and enable broader utilization of nationwide carcass data, thereby supporting the ongoing Hanwoo Performance and Progeny testing programs.

## APPLICATION OF GENOMIC PREDICTION IN HANWOO BEEF CATTLE

For HCs, whose genetic improvement over the past 40 years has been heavily focused on meat productivity [64], GS presents a strategic opportunity to expand selection goals and accelerate progress. The GP accuracies of the carcass traits of the Korean Hanwoo beef cattle are summarized in Table 1. However, the accuracy of GP using 50K SNP chips has shown variable performance depending on the reference population size, trait architecture, and marker density [1]. Table 2 presents a summary of the current investigations on GS using various GP methods and different genomic data for carcass traits in Hanwoo beef cattle.

The use of ssGBLUP, which integrates genotyped and non-genotyped individuals through joint modeling of pedigrees and genomic information, has become the gold standard in GS implementation [65]. In Hanwoo, this approach has been adopted in the national evaluation system following initial use of a multistep GBLUP model, leading to significantly improved predictive performance for carcass and body measurement traits [23,41,47]. Despite challenges in beef cattle, such as incomplete pedigrees, small progeny groups, and strong maternal effects [66], ssGBLUP has outperformed PBLUP in breeds such as Japanese Black, Angus, and Nellore [67–69]. Additionally, multi-trait models further increase prediction accuracy, particularly for correlated traits, as demonstrated by Jang et al [26], who reported higher prediction accuracy using a multi-trait ssGBLUP model for MS than both GBLUP and BayesR.WGBLUP and WssGBLUP have been developed to account for differential SNP effects. WGBLUP with nonlinear SNP weighting significantly increased prediction accuracy by 4.84% (BFT) and 2.70% (CWT) beyond that of GBLUP, but it has no added advantage over GBLUP for EMA and MS in HCs [49]. Similarly, when SNP weights and information from both genotyped and non-genotyped animals are incorporated, window-based weighting (WssGBLUP) significantly enhances accuracy, particularly for CWT (22%) and EMA (15%), with no accuracy gain for BFT and MS, indicating that these traits are influenced by major QTLs [53]. Moreover, the WssGBLUP model could outperform GBLUP for traits whose QTLs have moderate to large impacts [70]. Therefore, it is worth mentioning that WGBLUP or WssGBLUP is most beneficial for traits with large-effect QTLs, making it a trait-specific tool for increasing GP. Another study [51] reported that WGBLUP outperforms traditional GBLUP, where significant SNPs with distinct weights were considered to predict the accuracy of both the reproductive and carcass traits of HC. In addition, this study clarifies how important it is to include heterogeneity in SNP effect sizes in genomic assessments to increase prediction accuracy.

In HC, BayesR yielded higher prediction accuracies for CWT and BFT when the 50K SNP array was used [8]. BayesR also facilitated the identification of SNPs with substantial effects (ranging from 4.02% to 6.92%) on carcass traits, particularly within chromosomal regions BTA6 and BTA14, which are known to harbor influential QTLs [6]. This enhancement is attributed to the model’s ability to assign differential SNP effects, thereby more effectively capturing the influence of major QTLs. In comparative evaluations, both BayesR and GBLUP outperformed the traditional PBLUP, reinforcing the suitability of genomic models for selection in Hanwoo [30]. BayesC also outperformed both GBLUP and Bayesian LASSO for CWT, providing a 7% increase in accuracy, whereas performance for BFT, EMA, and MS remained comparable across methods [60]. These results suggest that CWT is regulated by a limited number of loci with large effects, whereas other carcass traits are more polygenic in nature. Further supporting evidence is provided by Lee et al [52], who applied a single-step Bayesian approach to a dataset comprising 988 genotyped and 1,438 non-genotyped Hanwoo individuals. This method yielded improved accuracy for CWT and demonstrated robust performance across all four carcass traits, underscoring its efficacy in integrating genomic and pedigree information. BayesB also produced higher heritability estimates for CWT and EMA, whereas GBLUP yielded better estimates for MS [33], suggesting differential model suitability depending on trait architecture. Similarly, BayesC outperformed GBLUP and Bayesian LASSO for CWT with a 7% gain, indicating the oligogenic nature of this trait [60]. Additionally, Bayesian models (e.g., MT-BayesCπ and mbBayesAB) have shown superior prediction accuracy over GBLUP for low-heritability reproductive traits in beef cattle [71,72] as well as in Hanwoo cows [73], highlighting the utility of Bayesian methods for traits influenced by major QTLs or with low heritability.

Recent studies have highlighted the increasing role of ML and deep learning in GP. In Chinese Simmental, ML algorithms such as SVR, KRR, RF, and AdaBoost. RT outperformed GBLUP for predicting the GEBVs of CWT, EMA, and live weight [74], whereas in Hanwoo, techniques such as MLR, NN, and PLSR were used to predict carcass traits from body measurements [27]. Similarly, Shahinfar et al [62], using data from 2,108 HCs, reported strong performance of ML methods for predicting MS and carcass traits, with SVM-SMO (support vector machine with sequential minimal optimization) achieving the best results. Table 3 summarizes different algorithms of ML and deep learning for the Hanwoo population. However, ML models do not always outperform traditional methods. For example, Srivastava et al [7] reported no consistent ML advantage over GBLUP, although XGBoost achieved higher accuracy for CWT and MS. These inconsistencies highlight that the performance of the ML model is highly context- and trait-dependent. Moreover, recent studies [10,75] have demonstrated the potential of deep learning for precision livestock management, achieving over 97% accuracy in identifying individual calves and HCs via muzzle pattern recognition, indicating strong applicability for real farms.

Furthermore, the integration of computer vision and digital phenotyping in GSs is an emerging field. Recently, Lee et al [76] introduced the F7 index, a novel marbling fineness score, which is based on the standard deviation of the marbling particle area across a grid and shows strong discriminatory power between BMSs 6–9 (p = 1.34×10^−27^). Such trait innovations can be integrated into GS programs, allowing breeders to target premium quality attributes more precisely. Another interesting study [54] in which the association of a genome-wide approach and GWAS resulted in a GP (called GWABLUP) yielded up to 10% and 13% more reliable GPs than did GBLUP for single and multi-trait analyses, respectively.

However, Hanwoo GP has largely focused on carcass and meat quality traits; similar trends have also been reported in other beef cattle. For instance, in Japanese black cattle, Onogi et al [67] observed higher predictive accuracies using ssGBLUP, particularly for CWT (0.84) and EMA (0.79), compared to Hanwoo [31]. Moreover, Ogawa et al [77] observed accuracies for CWT (0.85) and MS (0.60) using GBLUP with G matrix under different SNP densities, which are comparable with [60]. In contrast, the tropical beef breeds such as Nellore showed a lower predictive accuracy using GBLUP (0.30–0.38) and Bayesian methods (0.31–0.42) for the carcass traits (EMA, BFT, CWT) due to higher genetic diversity and limited reference populations [20]. Another study by Wang et al [78] investigated the prediction accuracy of GEBVs for growth, carcass, and meat quality traits in Chinese Simmental cattle, using BayesB and Elastic Net models, where the prediction accuracy (0.17 to 0.296) was comparatively lower than other beef breeds. In Angus, the high level of relatedness in training and validation animals substantially enhanced prediction accuracies for a range of carcass and production traits, ranging from 0.22–0.69 under low-relatedness to 0.38–0.85 under random splits with higher relatedness [79]. Similarly, in Hanwoo, low relatedness between reference and validation animals resulted in noticeably reduced accuracy for carcass traits, ranging from 0.21 to 0.45 [60]. Nevertheless, these comparisons indicate that advancements in Hanwoo genomic evaluation align well with global trends, while accuracy could be further enhanced by expanding reference populations.

## CHALLENGES AND OPPORTUNITIES

The success of GS depends on the ability to accurately predict GEBVs across generations, ideally without the need for repeated phenotyping once marker effects have been estimated. Importantly, the accuracy of GEBVs relies on reference population size, trait heritability, linkage disequilibrium, and QTLs [80]. Despite its proven utility in dairy cattle, the adoption and impact of GS in beef cattle, including HCs, have been relatively limited [81]. However, the adoption of GS in HC breeding offers substantial advantages in terms of prediction accuracy, reduced generation intervals, and genetic gains. It enables early, precise, and cost-effective improvement of key economic traits, supports the identification of major genes, and enables the development of targeted breeding strategies, thereby enhancing the genetic potential of the national herd. Moreover, the integration of genomic information into national performance testing programs and the expansion of reference populations can increase selection efficiency and long-term productivity. Thus, breeders should consider the supremacy of GS and adopt an appropriate prediction approach on the basis of their specific objectives. GP methods such as ssGBLUP, WGBLUP, WssGBLIP, and Bayesian have already proved their superiority in terms of carcass and meat quality traits over PBLUP. Despite these rewards, several challenges remain to be overcome. These include maintaining a balance among traits under selection, preserving genetic diversity, and ensuring the long-term sustainability of breeding programs. Addressing these issues requires advanced genomic approaches that consider population structure, gene-environment interactions, and the polygenic nature of complex traits [51]. Consequently, future research should be built on this framework to further exploit the incorporation of high-throughput phenotyping, multi-omics data, and robust statistical models in GP to maximize genetic enhancement while ensuring high-quality beef production. Furthermore, there is much room to investigate ML, such as the application of deep learning to HCs, as a novel idea in the beef industry, which will continue to propel the inventive advancement of Hanwoo breeding schemes.

## CONCLUSION

In conclusion, the genomic evaluation has significantly enhanced the breeding value prediction accuracy for economically important carcass traits. This review confirmed that all the GP models outperform the PBLUP method in the EBVs, and the integration of GBLUP and its extensions within the national evaluation system provides a powerful framework for accelerating genetic gain in the Hanwoo population. These GP approaches allow breeders to make early and more accurate selection decisions, particularly for traits like CWT and MS that are difficult to evaluate on live animals. The polycimaker must give strategic attention to developing the large and well-structured reference population and ensuring the routine gynotyping of breeding animals to sustain the accuracy of GP in Hanwoo. Over the next 5–10 years, the incorporation of whole-genome sequence data, the expansion of genomic evaluations to include reproductive and feed-efficiency traits, and the adaptation of AI-based prediction systems are expected to further strengthen the long-term sustainability and competitiveness of the Hanwoo breeding program.

## Figures and Tables

**Table 1:** Genomic prediction accuracies of carcass traits in Korean Hanwoo beef cattle

Model	Traits				Reference

	CWT	EMA	BFT	MS	
PBLUP	-	0.71	0.75	0.75	[82]
PBLUP	0.28–0.33	0.31–0.33	0.13–0.26	0.25–333	[52]
PBLUP	0.49	0.40	0.37	0.37	[49]
PBLUP	0.43	0.42	0.42	0.40	[41]
PBLUP	0.531	0.519	0.524	0.530	[44]
PBLUP	0.34	0.32	0.19	0.31	[53]
BLUP	0.575	0.566	0.572	0.578	[83]
PBLUP	0.53	0.53	0.52	0.54	[32]
PBLUP	0.434	0.445	0.435	0.431	[45]
PI	0.276	0.294	0.293	0.293	[45]
BLUP	0.380	0.373	0.338	0.366	[84]
ST-BLUP	0.33	0.30	0.25	0.28	[31]
ST-PBLUP	0.35	0.33	0.22	0.27	[23]
ST-BLUP	0.30	0.29	0.24	0.30	[47]
MT-BLUP	0.42	0.32	0.24	0.27	[31]
MT-PBLUP	0.36	0.33	0.22	0.26	[23]
MT-BLUP	0.35	0.33	0.29	0.30	[47]
GBLUP	-	0.72	0.76	0.76	[82]
GBLUP	0.33	0.31	0.25	0.25	[60]
GBLUP	0.74	0.67	0.62	0.65	[49]
GBLUP	-	-	-	0.19	[26]
GBLUP	0.451	0.437	0.421	-	[27]
GBLUP	0.58	0.51	0.48	0.47	[8]
GBLUP	0.58	0.51	0.48	0.47	[8]
GBLUP	0.799	0.780	0.787	0.810	[44]
GBLUP	0.672	0.662	0.662	0.693	[83]
GBLUP	0.634	0.659	0.619	0.627	[45]
GBLUP	0.743	0.728	0.737	0.765	[46]
GBLUP	0.73	0.68	0.68	0.74	[51]
ssGBLUP	0.42–0.46	0.40–0.42	0.29–0.35	0.32–0.37	[52]
ssGBLUP	0.44	0.44	0.43	0.41	[41]
ssGBLUP	-	-	-	0.27	[26]
ssGBLUP	0.70	0.39	0.41	0.43	[53]
ssGBLUP	0.73	0.71	0.70	0.74	[32]
ssGBLUP	0.749	0.733	0.769	0.768	[85]
ST-ssGBLUP	0.47	0.42	0.33	0.37	[31]
ST-ssGBLUP	0.78	0.73	0.66	0.70	[23]
ST-ssGBLUP	0.53	0.45	0.47	0.49	[47]
MT-ssGBLUP	0.56	0.44	0.33	0.36	[31]
MT-ssGBLUP	0.78	0.74	0.67	0.68	[23]
MT-ssGBLUP	0.56	0.48	0.48	0.49	[47]
WGBLUP	0.76	0.67	0.65	0.65	[49]
WGBLUP	0.59	0.50	0.50	0.47	[8]
WGBLUP	0.77	0.74	0.72	0.79	[51]
WssGBLUP	0.86	0.45	0.42	0.44	[53]
BayesC	0.40	0.31	0.26	0.25	[60]
BayesL	0.33	0.31	0.25	0.25	[60]
Bayesian	0.36–0.49	0.42–0.44	0.27–0.28	0.30	[52]
SSBR	0.41–0.52	0.41–0.43	0.34–0.35	0.36–0.37	[52]
BayesR	-	-	-	0.19	[26]
BayesR	0.61	0.51	0.50	0.47	[8]

CWT, carcass weight; EMA, eye muscle area; BFT, back fat thickness; MS, marbling score; PBLUP, pedigree-based best linear unbiased prediction; PI, pedigree index; ST-PBLUP, single-trait PBLUP; MT-PBLUP, multitrait PBLUP; GBLUP, genomic best linear unbiased prediction; ssGBLUP, single-step GBLUP; ST-ssGBLUP, single-trait single-step GBLUP; MT-ssGBLUP, multitrait single-step GBLUP; WGBLUP, weighted GBLUP; SSBR, single-step Bayesian.

**Table 2:** Summary of current investigations on genomic selection via various genomic prediction methods and different genomic data for carcass traits in Hanwoo beef cattle

Type of animals	Sample size (RD+TD)	Data collection from	Pedigree data	Genomic data	Traits	SNP density after QC	Models compared	References
Steers	1,183 (946+237)	HIC of the NACF	44,538	Illumina BovineSNP50 K and HD 777K Beadchips	CWT, BFT, EMA, MS	34,194	GBLUP, BayesLasso and BayesC	[60]
HC	7,991	KIAPE	38,731	Illumina Bovine SNP50k	CWT, BFT, EMA, MS	48,984	STPM, STGM, MTPM, MTGM	[25]
Steers, castrated males	10,215 (8,563+1,652)	KIAPE	29,176	Illumina 50K	CWT, BFT, EMA, MS	37,549	PBLUP, GBLUP and WGBLUP	[5]
Steers	1,151	Different commercial herds across South Korea	50,115	Illumina BovineSNP50K (959) and HD 777K (720)	CWT, BFT, EMA, MS	34,479	ST-BLUP, MT-BLUP, ST-ssGBLUP and MT-ssGBLUP	[31]
HC	1,160	HIC of the NACF	1.3 million	50K, DEG, 50k+DEG and Imputed WGS	MS	39,822, 321,614, 360,407 and 11,146,536	GBLUP, ssGBLUP, WssGBLUP and BayesR	[26]
HC	18,269 (15,228+3,041)	Hanwoo Research Institute in South Korea	32,807	Customized Hanwoo 50 K SNP Chip (Illumina), bovine HD BeadChip	CWT, BFT, REA and MS	45,567 and 560,826	ST-PBLUP, MT- PBLUP, ST- ssGBLUP-50 K, MT-ssGBLUP-50 K, ST- ssGBLUP-HD, and MT- ssGBLUP-HD	[23]
HC	13,717	4,000 farms in South Korea	-	Illumina 50K, Imputed WGS	CWT, BFT, EMA, MS	10,723,697	GBLUP, WGBLUP, and BayesR	[8]
Males	7,374	The Animal Genomics and Bioinformatics Division of the National Institute of Animal Science	67,802	Illumina BovineSNP50K BeadChip	CWT, BFT, EMA, MS	39,308	pedBLUP, ssGBLUP	[41]
Steers	12,635	KAIA	-	Illumina Bovine SNP50 BeadChip, Illumina Bovine HD BeadChip	CWT, BFT, EMA, IMF and WBSF	670,080 and 637,017	GBLUP (lm_777K), GBLUP (exp_777K), and GBLUP (exp_777K and text-mined SNPs jointly)	[27]
Mostly steers	9,568 (8,612+956)	Two different commercial populations	-	Hanwoo 50 k SNP Chip (Illumina, South Korea), imputed WGS	CWT, BFT, LMA and MS	37,712 and 26,936,924	GBLUP (GRM constructed on 50K, 50K+WGS and 50K+annotated different genomic regions with preselected SNPs)	[7]
Steers	5,622	4000 farms in South Korea	54,284	Illumina BovineSNP50K	CWT, BFT, EMA, MS	43,950	PBLUP. ssGBLUP, WssGBLUP	[53]
Cows	619	HIC of the NACF	-	Hanwoo 50K SNP analysis BeadChip	CWT, BFT, EMA, MS	-	BLUP and GBLUP	[83]
Steers	9302	Korea Beef Improvement Institute	-	Illumina Bovine 50K v.3 SNP chip	CWT, BFT, EMA, MS	41,496	GBLUP and Bayes B	[33]
Cows and steers	8,023,666	Nonghyup livestock farms in Korea	13,534,295	Affymetrix Axiom Bovine 60 K chip, Illumina Bovine 50 K chip, customized Hanwoo 50 K chip	CWT, BFT, EMA, MS	-	single-step marker effect model and conventional BLUP model	[57]
HC	94,969	-	74,730	Hanwoo 50 K SNP beadchip.	CWT, BFT, EMA, MS	45,548	ssGBLUP	[85]
HC	18,499	Gyeonggi, Gangwon, Chungbuk, and Gyeongnam	1,740	Hanwoo 50K SNP Analysis BeadChip	CWT, BFT, EMA, MS	45,548	GBLUP	[46]
Cows	1,544	KAIA	-	Illumina Bovine 50K SNP Chip	CWT, BFT, EMA, MS, AFC, CI, GL, NAIPC	41,445	GBLUP and WGBLUP	[51]

RD, reference data; TD, testing data; QC, quality control; HIC, Hanwoo improvement center; NACF, national agricultural cooperative federation; CWT, carcass Weight; BFT, backfat thickness; EMA, eye muscle area; MS, marbling score; GBLUP, genomic best linear unbiased prediction; HC, Hanwoo cattle; KIAPE, Korean Institute for Animal Products Quality Evaluation; STPM, single-trait pedigree model; STGM, single-trait genomic model; MTPM, multitrait pedigree model; MTGM, multitrait genomic model; WGBLUP, weighted GBLUP; ST-PBLUP, single-trait PBLUP; MT-PBLUP, multitrait PBLUP; ST-ssGBLUP, single-trait single-step GBLUP; MT-ssGBLUP, multitrait single-step GBLUP; DEG, differentially expressed genes; WGS, whole genome sequence data; ssGBLUP, single-step GBLUP; WssGBLUP, weighted ssGBLUP; REA, rib eye area; pedBLUP, pedigree BLUP; KAIA, Korea Animal Improvement Association; IMF, intramuscular fat; WBSF, Warner–Bratzler shear force; (lm_777K) and (exp_777K), genomic model using genomic relationship matrix constructed by the imputed 777K; LMA, longissimus muscle area; AFC, age at first calving; CI, calving interval; GL, gestation length; NAIPC, number of artificial inseminations per conception.

**Table 3:** Application of different machine learning and deep learning algorithms to the Hanwoo population

Application	Data type	ML/DL methods	Key outcomes	Reference
Carcass & marbling	Genomic+phenotypic	LR, MLP, MT, RF, SMO, SMO+SMOTE	- Best algorithm: SMO - Highest accuracy: CWT (0.95)	[62]
Cold CWT	Body measurements	MRA, PLSR, ANN	- Best algorithm: ANN - Highest accuracy: 0.92	[86]
Marbling grading	Images of sirloin	CNN	- Training accuracy: 100% - Test accuracy: 86%	[37]
Weight	Image+measurements	MLR	Weight estimation feasible	[87]
% IMF	Image	CNN	98.2% accuracy	[88]
CWT, MS, BFT, EMA	Genomic+phenotypic	RF, XGBoost, SVM, GBLUP	- CWT & MS: XGBoost>GBLUP>SVM>RF BFT & EMA: GBLUP>SVM>RF>XGBoost	[7]
Meat color, pH, WHC, shear force, grilling loss	Phenotypic	LR, DT	Effectively predict beef quality traits	[89]
Live body weight	Images+measurements	LightGBM, MLP, k-NN, TabNet, FT-transformer	- High prediction accuracy - algorithm: LightGBM regression	[90]
Body weight	3D mesh	RF, CatBoost, LightGBM, XGBoost, PR	Best algorithm: RFR	[91]
Behavior	Video	DL	Supports automated, spatiotemporal cattle behavior analysis	[92]
Multiple quantitative traits	Genotypic+phenotypic	deepGBLUP, GBLUP, Bayesian	Best algorithm: deepGBLUP	[63]
Estrus/Heat detection	Sensor/IMS	ARM-based IMS	Promising estrus detection	[93]
Muzzle-based ID	Image+meta	EfficientNet V2 optimized with SGD, RMSProp, Adam, Lion	- High ID accuracy - Best validation accuracy: EfficientNet v2 small+Lion optimizer (0.970) Most stable performance: EfficientNet v2 small+RMSProp optimizer	[75]
SNP subsets selection	Genotypic+phenotypic	GBM, XGBoost, GWAS	- Highest accuracy (all SNPs): GBM (0.79)>XGBoost (0.77)>GWAS (0.76) - Theoretical accuracy: GWAS (0.93)>GBM (0.86)≈XGBoost (0.85)	[94]
Beef authenticity	Spectral	RF, SVM, LoR, GB, NN, KNN, DT, NB, LDA	- Best model: RF (AUC = 0.8826)	[95]

ML, machine learning; DL, deep learning; LR, linear regression; MLP, multilayer perceptron; MT, model tree; RF, random forest; SMO, support vector machine with sequential minimal optimization; SMOTE, synthetic minority oversampling technique; CWT, carcass weight; MRA, multiple regression analysis; PLSR, partial least squares regression; ANN, artificial neural network; CNN, convolutional neural network; MLR, multiple linear regression; % IMF, intramuscular fat percentage; MS, marbling score; BFT, back fat thickness; EMA, eye muscle area; SVM, support vector machine; GBLUP, genomic best linear unbiased prediction; pH, hydrogen ion concentration; WHC, water boosting holding capacity; DT, decision tree; LightGBM, light gradient boosting machine; k-NN, k-nearest neighbors; XGBoost, extreme gradient boosting; PR, polynomial regression; RFR, random forest regression; deepGBLUP, joint deep learning and genomic best linear unbiased prediction; IMS, intelligent monitoring system; ARM, augmented recognition model; SGD, stochastic gradient descent; GBM, gradient boosting machine; GWAS, genome wide association study; LoR, logistic regression; GB, gradient boosting; NN, neural network; NB, naive bayes; LDA, linear discriminant best linear unbiased prediction; AUC, area under the curve.

## Data Availability

Upon reasonable request, the datasets of this study can be available from the corresponding author.
